# *In vivo* fluorescence correlation spectroscopy analyses of FMBP‐1, a silkworm transcription factor

**DOI:** 10.1002/2211-5463.12026

**Published:** 2016-01-27

**Authors:** Motosuke Tsutsumi, Hideki Muto, Shohei Myoba, Mai Kimoto, Akira Kitamura, Masakatsu Kamiya, Takashi Kikukawa, Shigeharu Takiya, Makoto Demura, Keiichi Kawano, Masataka Kinjo, Tomoyasu Aizawa

**Affiliations:** ^1^ Faculty of Advanced Life ScienceHokkaido UniversitySapporoJapan; ^2^ Biomedical Research Support CenterNagasaki University School of MedicineNagasaki, Japan; ^3^ Faculty of ScienceHokkaido UniversitySapporoJapan; ^4^ Chitose Institute of Science and TechnologyChitose, Japan

**Keywords:** DNA‐binding protein, fluorescence correlation spectroscopy, one score and three peptide repeat domain, silkworm, transcription factor

## Abstract

Fibroin modulator‐binding protein 1 (FMBP‐1) is a silkworm transcription factor that has a unique DNA‐binding domain called the one score and three amino acid peptide repeat (STPR). Here we used fluorescence correlation spectroscopy (FCS) to analyze the diffusion properties of an enhanced green fluorescent protein‐tagged FMBP‐1 protein (EGFP‐FMBP‐1) expressed in posterior silk gland (PSG) cells of *Bombyx mori* at the same developmental stage as natural FMBP‐1 expression. EGFP‐FMBP‐1 clearly localized to cell nuclei. From the FCS analyses, we identified an immobile DNA‐bound component and three discernible diffusion components. We also used FCS to observe the movements of wild‐type and mutant EGFP‐FMBP‐1 proteins in HeLa cells, a simpler experimental system. Based on previous *in vitro* observation, we also introduced a single amino acid substitution in order to suppress stable FMBP‐1‐DNA binding; specifically, we replaced the ninth Arg in the third repeat within the STPR domain with Ala. This mutation completely disrupted the slowest diffusion component as well as the immobile component. The diffusion properties of other FMBP‐1 mutants (e.g. mutants with N‐terminal or C‐terminal truncations) were also analyzed. Based on our observations, we suggest that the four identifiable movements might correspond to four distinct FMBP‐1 states: (a) diffusion of free protein, (b) and (c) two types of transient interactions between FMBP‐1 and chromosomal DNA, and (d) stable binding of FMBP‐1 to chromosomal DNA.

**Abbreviations**
ACF: autocorrelation function
FCS: fluorescence correlation spectroscopy
FMBP‐1: fibroin modulator‐binding protein‐1
STPR: one score and three peptide repeat

Silk glands of silkworm *Bombyx mori* larvae are among the most famous protein‐producing organs in scientific literature. When cocoon formation begins, the gross weight of these silk glands accounts for nearly 40% of larval weight, and these organs store huge amounts of silk protein. The larval silk gland of *B. mori* is distinctly divided into the anterior silk gland (ASG), the middle silk gland (MSG), and the posterior silk gland (PSG) based on structural and functional criteria. A silk gland comprises about 600 cells, and each cell grows without cell division. Thus, each cell becomes very large; moreover, each nucleus becomes polyploid and develops a dendritic form 1, 2.

Fibroin is the main component of silk protein, and it consists of a heavy (H) chain, light (L) chain, and P25. Throughout larval development, fibroin is expressed in the PSG during every feeding stage, but not during any molting stage 2. Such tissue‐ and temporal‐specific expression of the fibroin protein is considered to be precisely controlled by a number of transcription factors (TFs). Several fibroin TFs such as BMFA 3, SGFB 3, 4, Fkh/SGF‐1 5, 6, 7, SGF‐2 6, POU‐M1/SGF‐3 6, 8, 9, Bmsage 10, and FMBP‐1 (fibroin modulator‐binding protein‐1) 9, 11 have been identified. However, a comprehensive picture of fibroin gene expression is lacking.

FMBP‐1 is a recently identified TF that specifically binds to a 9 bp AT‐rich motif (5′‐ATNTWTNTA‐3′) in upstream and intronic promoter elements of the gene encoding the fibroin H chain 9. FMBP‐1 comprises 218 amino acid residues and is divided into several distinctive domains on the basis of amino acid sequence. Notably, the C‐terminal half has a unique structure that comprises four tandem repeats of a 23‐residue domain, known as the one score and three amino acid peptide repeat (STPR) domain, which acts as a DNA‐binding domain in FMBP‐1 11. Various properties of FMBP‐1 have been determined via biochemical or structural biological techniques 12, 13, 14. For example, analyses involving nuclear magnetic resonance, circular dichroism, and limited digestion have shown that the STPR domain adapts a quite rigid, helix‐rich structure when bound to DNA, but forms a flexible structure in the absence of DNA 13. Mutational analysis of the STPR domain demonstrated that each salt bridge between the fourth glutamic acid residue and the ninth arginine residue of each repeat is important to the rigid structure adopted by FMBP‐1 in the DNA‐bound state. However, the dynamics between FMBP‐1 and DNA *in vivo* remain unknown.

Here, we used fluorescence correlation spectroscopy (FCS) techniques to assess these dynamics *in vivo*. FCS is a powerful method used to observe many kinds of molecular dynamics and interactions in aqueous conditions with single‐molecule sensitivity 15, 16, 17. FCS measures fluorescence intensity fluctuation caused by Brownian motion of fluorescent molecules in a small volume element (a femtoliter‐volume in the shape of a prolate spheroid) that is generated in the sample through the confocal arrangement of optical elements. Then, an autocorrelation curve is mathematically extracted from the measured fluorescence fluctuation; this temporal autocorrelation curve is then compared to autocorrelation functions (ACFs) of several diffusion models by curve fitting. Comparisons between the extracted functions and the models are used to determine (a) the diffusion time, (b) the number of diffusion components, and (c) the relative abundance of each diffusion component. Recently, FCS techniques have been used to study several TFs in cultured cells 18, 19, 20. Furthermore, FCS‐based quantitative techniques have been used to analyze homeodomain‐DNA interactions in *Drosophila* salivary gland cells 21. These studies and findings indicate that FCS analysis could be used to study FMBP‐1 dynamics in silk gland cells.

In this study, we utilized FCS to analyze the diffusion dynamics of FMBP‐1 in the PSGs of *B. mori* fifth instar larvae, which represent the tissue and larval stage positive for endogenous FMBP‐1.

## Results

### Laser scanning microscopy observations of FMBP‐1 in PSG cells

**Figure 1:**
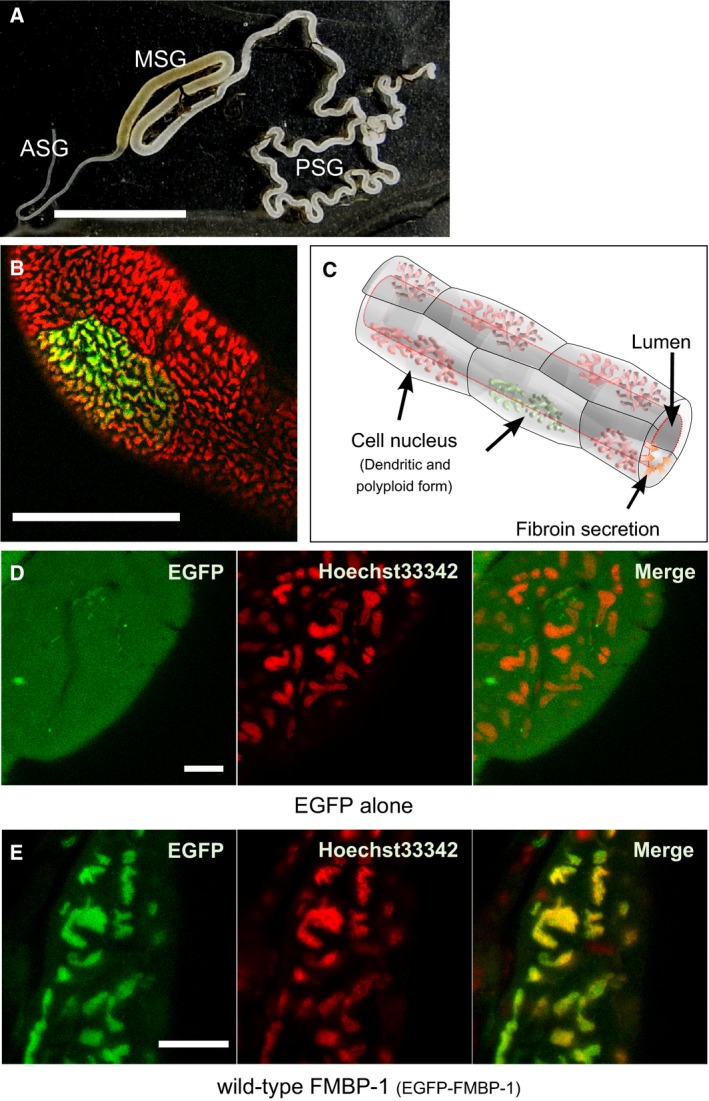
Localization of EGFP‐FMBP‐1 in the posterior silk gland cells. (A) The structure of a silk gland dissected from a fifth instar *Bombyx mori* larvae. The scale bar is 5 mm. The silk gland is distinctly divided into an anterior silk gland (ASG), a middle silk gland (MSG), and a posterior silk gland (PSG). Isolated PSGs were used for all fluorescence correlation spectroscopy (FCS) measurements. (B) Confocal overview image of PSG cells. The scale bar is 500 μm. Each cell nucleus was stained with Hoechst33342 (red), and only a few cells expressed the transgene (green). The PSG is a tubular organ consisting of alternately facing cells. Each cell has a dendritic polyploid nucleus. (C) Schematic image of the typical structure of PSG cells. This image was drawn based on the image in (B). (D) Confocal images of a PSG cell transiently expressing EGFP alone. The scale bar is 50 μm. (E) Confocal image of a PSG cell transiently expressing wild‐type EGFP‐FMBP‐1. The scale bar is 50 μm. These confocal images were taken with Carl Zeiss LSM510 microscope (EGFP fluorescence, Ex. 488 nm, Em. 505–540 nm. Hoechst 33342 [nuclear stain] fluorescence, Ex. 405 nm, Em. 420–480 nm).

To mimic *in vivo* conditions as closely as possible while observing FMBP‐1 mobility, we used transfected cells from PSGs of fifth instar *B. mori* larvae, which transiently express a fusion protein (EGFP‐FMBP‐1) comprising an EGFP tag and full‐length FMBP‐1 sequence. As controls, PSG cells that expressed EGFP alone were prepared in parallel. The PSG is a tubular tissue that constitutes the posterior half of the silk gland, and is the site of fibroin protein production (Fig. 1A). A confocal image of a Hoechst‐stained EGFP‐expressing PSG is shown at low magnification in Fig. 1B. The alternating and consecutive arrangement of the semicylindrical cells forms the lumen of the PSG (Fig. 1C). In PSGs that expressed EGFP alone, fluorescence was observed throughout the cell and was not exclusively localized to the cell nuclei (Fig. 1D). In contrast, PSGs that expressed EGFP‐FMBP‐1 showed clear nuclear localization of the EGFP signal (Fig. 1E).

### FCS analyses of EGFP signals in PSG cells

**Figure 2:**
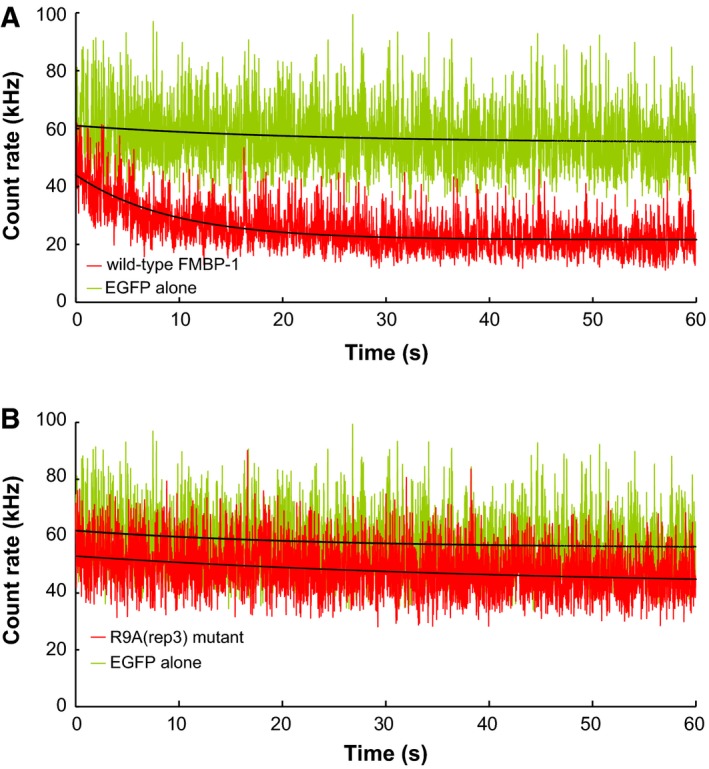
Fluorescence fluctuation from EGFP‐FMBP‐1 in posterior silk gland (PSG) cells. Sequential record data of fluorescence intensity of (A) wild‐type FMBP‐1 and (B) R9A(rep3) mutant (red). The intensity of EGFP alone with similar expression level was used as a control (green). Black curves lying on each fluctuation record indicate the fitting curve of photobleaching drawn by the fitting Eqn (2) described in the Materials and Methods.

Next, we measured the fluorescence fluctuations of the EGFP‐FMBP‐1 signal in the nuclei of PSG cells. The fluorescence intensity of EGFP‐FMBP‐1 showed obvious decay during the first few tens of seconds of each measurement (Fig. 2A). In contrast, the fluorescence intensity of EGFP alone remained almost constant. The photobleaching of EGFP‐FMBP‐1 likely resulted from the immobilization of a fraction of EGFP‐FMBP‐1 molecules due to DNA binding, whereas for EGFP alone, free diffusion ensured that the resulting signal did not decay as rapidly.

**Figure 3:**
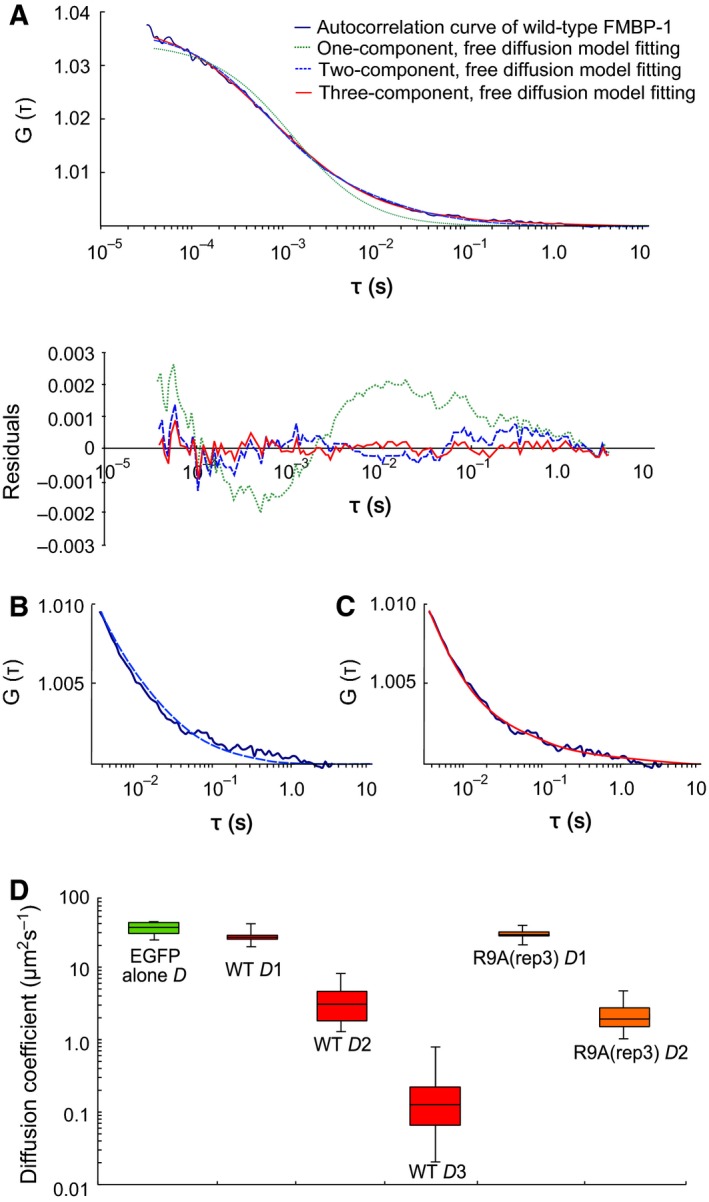
Fluorescence correlation spectroscopy (FCS) analyses of EGFP‐FMBP‐1 in the posterior silk gland (PSG) cells. (A) ACF of wild‐type FMBP‐1 FCS (navy blue line) with various fitting models: the one‐component free diffusion model (green dot line), the two‐component free diffusion model (blue dot line), and the three‐component free diffusion model (red line). A graph of the residuals is shown below the images. (B, C) Enlarged views of latter half of (A). Fitting curves of the two‐component, free diffusion model and three‐component, free diffusion model are shown in (B) and (C), respectively. (D) Box plot of diffusion coefficient of EGFP alone (green), three diffusion coefficients of wild‐type FMBP‐1 (WT, red) and two diffusion coefficients of R9A(rep3) mutant (orange). The whiskers show the minimum and maximum value of each coefficient. These diffusion coefficients were converted from diffusion times, which were measured via FCS, the conversion formula (4) described in the Materials and Methods was used for all conversions.

For correct FCS analysis, the observable photobleaching time period of EGFP‐FMBP‐1 was removed from the ensemble calculation to extract the ACF. Then, each of three free diffusion models (one‐, two‐. and three‐component) were used to fit the autocorrelation curves (Fig. 3A–C). The residuals showed the systematic deviations for each model. One‐ and two‐component fits had larger χ^2^ values than did the three‐component fits. Akaike's information criteria (AIC) and *F*‐tests were also used to compare the probabilities associated with the respective models; both analyses strongly indicated that the three‐component model most closely fit the data (Tables S1 and S2). Furthermore, inclusion of a fourth diffusion component did not improve the fit, nor did it result in reasonable diffusion parameters. In addition, three anomalous diffusion models (a one‐component and two‐component models with one anomalous and one free component or with both components anomalous) were also tested. The residuals of the curve fitting with the one‐component anomalous diffusion model were significantly larger than those of the three‐component free diffusion model. However, the residuals of the curve fitting with the other two anomalous diffusion models were slightly smaller than that of three‐component free diffusion model. Nevertheless, the preciseness of the fitting parameters was also an important factor in choosing the best‐fitting model 22. However, the diffusion parameters that were determined by both two‐component anomalous diffusion models varied greatly (maximum standard deviation of the parameters were larger than 200%); consequently, the interpretation of each diffusion component was very difficult (Table S3). For reference, a three‐component anomalous diffusion model was also tested for fit (Table S3). The diffusion parameters obtained by the three‐component anomalous diffusion model also varied greatly. Therefore, the autocorrelation curves of EGFP‐FMBP‐1 were accounted for by the three‐component free diffusion model. The EGFP‐FMBP‐1 diffusion parameters measured in the PSG cells are shown in Table 1.

**Table 1:** Diffusion parameters of EGFP‐FMBP‐1 in posterior silk gland cells

	1st component		2nd component		3rd component		Bleach	*n*
	*F*_1_ (%)	τ_1_ (μs)	*F*_2_ (%)	τ_2_ (ms)	*F*_3_ (%)	τ_3_ (ms)		
EGFP alone	100	266.7 ± 53.4					−	13
Wild‐type	45.1 ± 14.0	343.2 ± 59.8	41.4 ± 7.6	3.28 ± 1.69	13.5 ± 9.5	98.2 ± 88.3	+	29
R9A(rep3)	76.9 ± 4.0	339.8 ± 48.7	23.1 ± 4.0	5.99 ± 2.99			−	16

These values were derived by curve fitting of the autocorrelation function via the optimum fitting model for each sample. The ‘±’ in each column indicates standard deviation (SD). The ‘+’ and ‘–’ in the ‘Bleach’ column indicate the presence or absence, respectively, of observable photobleaching.

The diffusion times determined from the FCS measurements indicated the transit times of fluorescent particles in a confocal volume (here, approximately 400 nm in diameter and 2000 nm in height). The diffusion coefficient of the 1st component of EGFP‐FMBP‐1 was similar to that of EGFP alone (Fig. 3D). In contrast, the diffusion coefficients of the 2nd and 3rd components of EGFP‐FMBP‐1 were significantly smaller than those of EGFP alone. These results indicated that EGFP‐FMBP‐1 molecules exhibited three different diffusion modes and one immobile (photobleaching) state *in vivo*.

### FCS analyses of EGFP‐FMBP‐1 mutants in PSG cells

Previous mutational analyses had shown that substitution of the ninth arginine with an alanine in repeat 3 of the STPR domain (R9A(rep3)) most clearly disrupted the rigid helix‐rich domain conformation and reduced the ability of FMBP‐1 to stably bind DNA 13. To further characterize the four putative components (three diffusion components and one immobile component) of FMBP‐1 dynamics, an R9A(rep3) EGFP‐FMBP‐1 mutant was compared to wild‐type EGFP‐FMBP‐1 in PSG cells.

**Figure 4:**
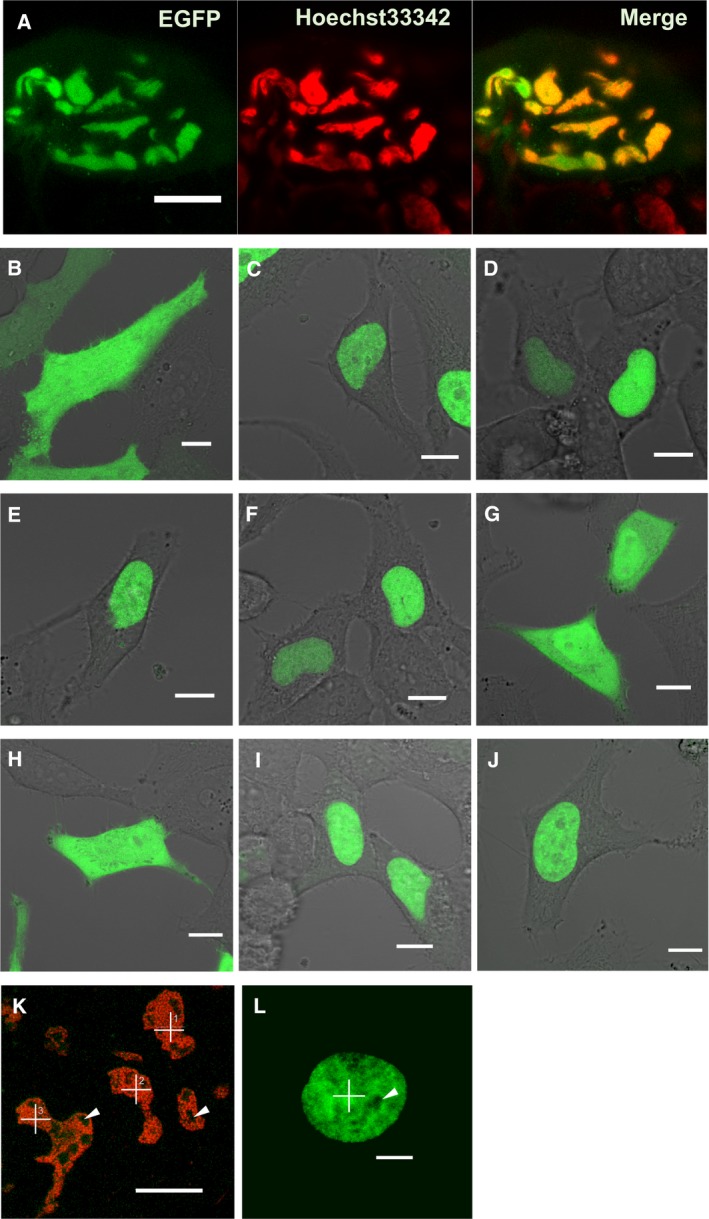
Nuclear localization of each EGFP‐FMBP‐1 mutant. (A) Confocal image of a posterior silk gland (PSG) cell transiently expressing R9A(rep3) mutant. The image was taken with a Carl Zeiss LSM510 microscope (EGFP fluorescence, Ex. 488 nm, Em. 505–540 nm. Hoechst 33342 (nuclear stain) fluorescence, Ex. 405 nm, Em. 420–480 nm). The scale bar is 50 μm. (B–J) Confocal images of HeLa cells expressing (B) EGFP alone, (C) wild‐type FMBP‐1, (D) R9A(rep3), (E) ΔCtail, (F) ΔC‐rep4, (G) ΔC‐STPR, (H) ΔC‐HBR, (I) ΔN‐HBR, or (J) ΔN‐AR mutant were taken with a Carl Zeiss LSM510 microscope (EGFP fluorescence, Ex. 488 nm, Em. 505–540 nm). The scale bar is 10 μm. (K) Measurement points for fluorescence correlation spectroscopy (FCS) analyses in a PSG cell expressing wild‐type FMBP‐1. The image was taken in the same manner as the images in (A). The scale bar is 20 μm. (L) Measurement point for FCS analyses in a HeLa cell expressing wild‐type FMBP‐1. The image was taken in the same manner as image (B–J). The scale bar is 5 μm. In the image (K) and (L), cross marks show the measurement points for FCS, and arrowheads indicate the nucleoli.

**Figure 5:**
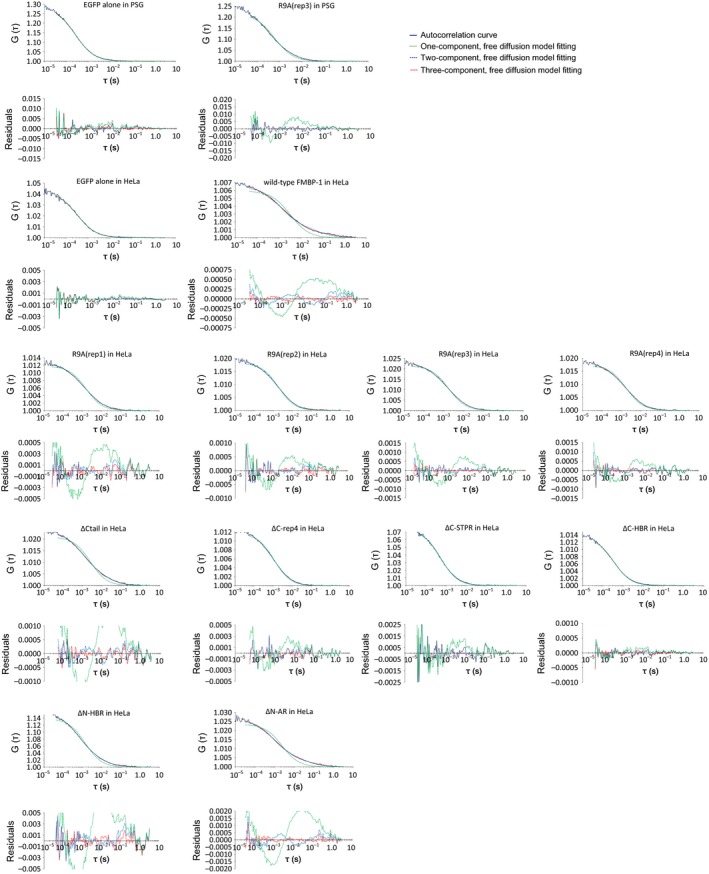
Curve fitting for various mutants of EGFP‐FMBP‐1. Representative autocorrelation function (ACFs) of various mutants of EGFP‐FMBP‐1 (navy blue line) with three fitting models: the one‐component, free diffusion model (green dot line); the two‐component, free‐diffusion model (blue dot line); and the three‐component, free diffusion model (red dot line). A graph of the residuals is shown below each ACF graph. In this study, the number of diffusion components was judged by fitting residuals and appropriateness of diffusion parameters, which were calculated by the curve fitting. As a result, the three‐component model was found to be appropriate for the wild‐type FMBP‐1 (in both posterior silk gland (PSG) cells and HeLa cells), R9A(rep1), ΔCtail, ΔN‐HBR and ΔN‐AR mutants. The two‐component model was found to be appropriate for the R9A(rep2), R9A(rep3) (in both PSG cells and HeLa cells), R9A(rep4), ΔC‐rep4 and ΔC‐STPR mutants. The one‐component model was found to be appropriate for EGFP alone (in both PSG cells and HeLa cells) and the ΔC‐HBR mutant.

**Figure 6:**
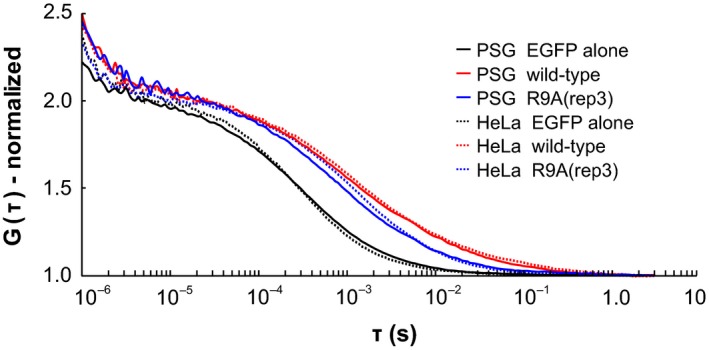
Autocorrelation curve of EGFP‐FMBP‐1. Autocorrelation curves based on measurements from posterior silk gland (PSG) cells (solid lines) or HeLa cells (dotted lines). Each autocorrelation curve represents the average of all measurements taken from each sample; the curves were normalized to have the same amplitude.

The R9A(rep3) mutant, like wild‐type FMBP‐1, localized to the nucleus of each PSG cell (Fig. 4A). However, the mutant did not exhibit photobleaching during FCS measurements (Fig. 2B). In addition, the two‐component diffusion model was appropriate for curve fitting of the autocorrelation curve of the R9A(rep3) mutant (Fig. 5); here, inclusion of a third diffusion component did not improve the fit. The results of the fitting analysis showed such a low amplitude that no reliable diffusion time could be determined. Normalized autocorrelation curves derived from all measured data from wild‐type FMBP‐1 or the R9A mutant were compared. As the R9A(rep3) mutant and wild‐type FMBP‐1 proteins differed by only one amino acid residue, the two proteins should have similar molecular weights; however, their autocorrelation curves clearly differed (Fig. 6). Thus, this change in the autocorrelation curves should represent changes in the interactions between FMBP‐1 and other molecules in the nuclei. The diffusion parameters for the R9A(rep3) mutant measured in the PSG cells are shown in Table 1. Each diffusion time obtained from curve fitting corresponded to the 1st or 2nd component of the wild‐type FMBP‐1 (Table 1 and Fig. 3D). In other words, the R9A(rep3) mutant simultaneously lost both the 3rd diffusion component and the immobile component. Taken together, this result and previous findings indicated that these two components were dependent on the stable binding of FMBP‐1 to DNA.

### Observation of wild‐type EGFP‐FMBP‐1 in HeLa cells

To further examine each diffusion component and the role of each domain of FMBP‐1 in more detail, it was necessary to observe various FMBP‐1 mutants. However, observing such mutants in PSG cells was difficult because sample preparation using the tungsten gun method was complicated. Additionally, measurement success rates were low; in particular, low transformation efficiency with PSG cells caused a decrease in measurement success rates. Furthermore, the strong intrinsic fluorescence of PSG cells and the instability of the fluorescence intensity of EGFP in these cells made it difficult to efficiently measure diffusion properties. Therefore, we analyzed the diffusion of EGFP‐tagged FMBP‐1 mutants in HeLa cells, which represent a simpler experimental system. HeLa cells were also used in a previous analysis of the ZNF821 protein (an ortholog of the STPR domain in humans) that was performed by our group 23.

**Figure 7:**
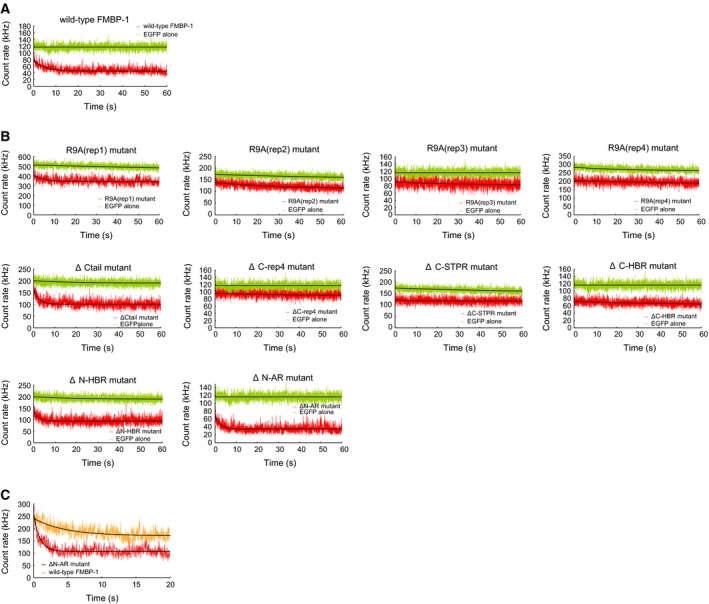
Fluorescence fluctuation of EGFP‐FMBP‐1 mutants. Sequential record data of fluorescence intensity with (A) wild‐type EGFP‐FMBP‐1 or (B) various FMBP‐1 mutants in HeLa cells (red). The initial intensity record data of EGFP alone (green) was compared as the control, as in Fig. 2. (C) Comparison between ΔN‐AR (red) and wild‐type EGFP‐FMBP‐1 (orange) with regard to photobleaching ratio. The initial intensity levels of both fluctuations were almost identical. Black curves lying on each fluctuation record indicate the fitting curve of photobleaching drawn by the fitting Eqn (2) described in the Materials and Methods.

**Figure 8:**
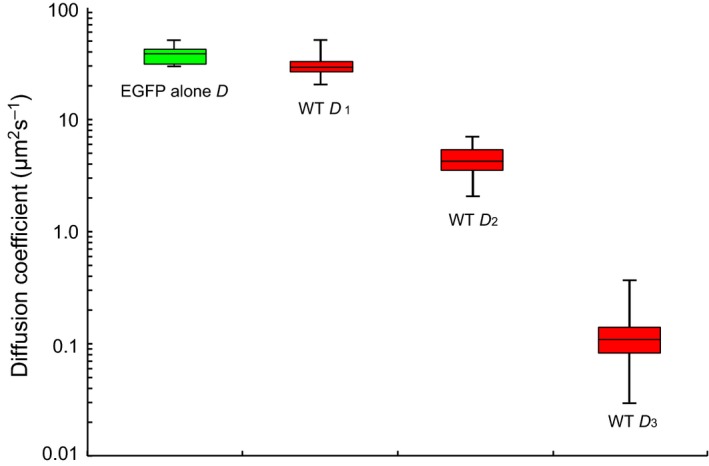
Diffusion coefficient of wild‐type EGFP‐FMBP‐1 in HeLa cells. Box plot of the diffusion coefficient of EGFP alone (green) and three diffusion coefficients for wild‐type EGFP‐FMBP‐1 (WT, red). The whiskers show the minimum and maximum value of each coefficient. These diffusion coefficients were converted from the diffusion times determined via fluorescence correlation spectroscopy (FCS) analyses and calculated via the conversion formula (4) (shown in Materials and Methods).

First, we determined whether each diffusion movement of wild‐type EGFP‐FMBP‐1 observed in PSG cells could be reproduced in HeLa cells. EGFP‐FMBP‐1 showed clear nuclear localization in confocal images of HeLa cells, as it had in PSG cells (Fig. 4B,C). The EGFP‐FMBP‐1 measured in HeLa cell nuclei also showed photobleaching during the early stage of each measurement (Fig. 7A). Both normalized autocorrelation curves of EGFP alone and of EGFP‐FMBP‐1 showed shapes similar to those seen with PSG cell measurements (Fig. 6). The three‐component diffusion model was also appropriate for the curve fitting of data from EGFP‐FMBP‐1 in HeLa cell nuclei (Fig. 5). The diffusion times of wild‐type FMBP‐1 are listed in Table 2. The range of diffusion times were very similar to those observed in the PSG cells (Fig. 8). These results confirmed that the diffusion movements of EGFP‐FMBP‐1 in HeLa cells accurately reproduced those in PSG cells.

**Table 2:** Diffusion parameters of various EGFP‐FMBP‐1 mutants in HeLa cells

	1st component		2nd component		3rd component		Bleach	*n*
	*F*_1_ (%)	τ_1_ (μs)	*F*_2_ (%)	τ_2_ (ms)	*F*_3_ (%)	τ_3_ (ms)		
EGFP alone	100	278.2 ± 19.5					−	15
Wild‐type	33.9 ± 7.7	335.1 ± 52.8	52.8 ± 5.4	2.43 ± 0.67	13.3 ± 6.9	112.1 ± 79.6	+	27
R9A(rep1)	36.9 ± 4.8	355.8 ± 42.8	59.3 ± 4.5	2.51 ± 0.78	3.8 ± 1.8	209.1 ± 203.3	+	8
R9A(rep2)	44.2 ± 5.2	355.8 ± 37.0	55.8 ± 5.2	3.47 ± 0.51			−	12
R9A(rep3)	40.9 ± 6.8	354.8 ± 85.2	59.1 ± 6.8	3.20 ± 1.30			−	15
R9A(rep4)	45.0 ± 7.3	390.6 ± 85.9	55.0 ± 7.3	4.21 ± 0.86			−	7
ΔCtail	33.0 ± 4.3	330.9 ± 45.7	55.2 ± 6.4	2.67 ± 0.81	11.8 ± 4.9	77.3 ± 44.3	+	18
ΔC‐rep4	50.4 ± 11.5	357.7 ± 77.0	49.6 ± 11.5	2.85 ± 1.19			−	14
ΔC‐STPR	80.3 ± 13.6	326.2 ± 64.5	19.7 ± 13.6	1.70 ± 0.52			−	10
ΔC‐HBR	100	345.8 ± 36.8					−	21
ΔN‐HBR	33.5 ± 4.4	271.7 ± 36.9	57.4 ± 3.7	1.65 ± 0.42	9.1 ± 4.5	33.2 ± 14.0	+	7
ΔN‐AR	36.9 ± 3.9	292.7 ± 41.5	45.5 ± 5.4	2.29 ± 0.47	17.6 ± 5.6	60.6 ± 33.6	+	10

These values were derived by curve fitting of the autocorrelation function with the optimum fitting model for each sample. The ‘±’ in each column indicate SD. The ‘+’ and ‘–‘ in the ‘Bleach’ column indicate the presence or absence, respectively, of observable photobleaching.

### FCS analyses of EGFP‐FMBP‐1 mutants in HeLa cells

**Figure 9:**
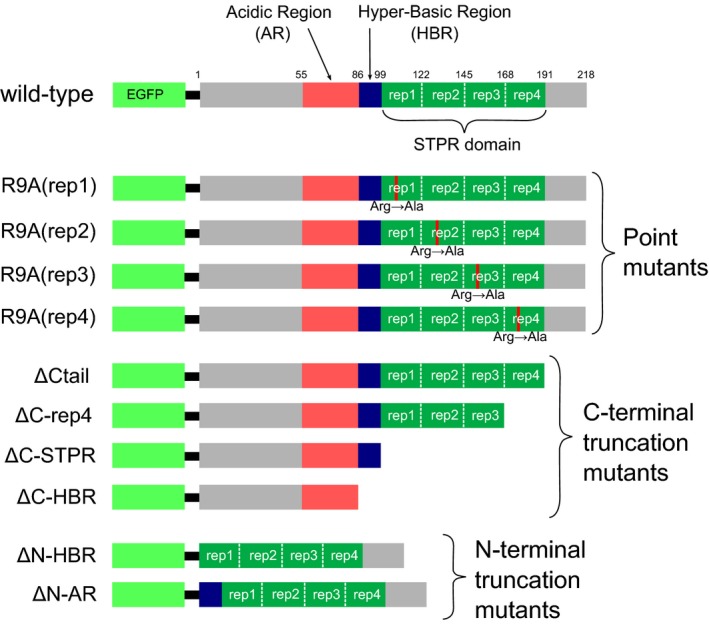
Representation of mutant forms of EGFP‐FMBP‐1. Schematic representation of EGFP‐FMBP‐1 molecules used in this study. The enhanced green fluorescent protein (EGFP) sequences were fused in‐frame to the 5′ end of the FMBP‐1 sequences. Substitution mutants were constructed such that Arg9 of each score and three peptide repeat (STPR) repeat was separately replaced with Ala to generate the R9A(rep1), R9A(rep2), R9A(rep3), and R9A(rep4) mutants. The C‐terminal truncation mutants were as follows: ΔCtail mutant, truncated C‐terminal tail; ΔC‐rep4 mutant, truncated C‐tail and fourth repeat of STPR domain; ΔC‐STPR mutant, truncated C‐tail and all repeats of STPR domain; and ΔC‐HBR mutant, truncated from C‐tail to hyper basic region (HBR). Each was used for fluorescence correlation spectroscopy (FCS) measurements. The N‐terminal truncation mutants were as follows: ΔN‐HBR mutant, truncated N‐terminal region from N terminus to HBR and ΔN‐AR mutant, truncated from N terminus to acidic region (AR).

Next, we constructed mutant versions of EGFP‐FMBP‐1 and transfected HeLa cells with each mutant separately (Fig. 9). Substitution mutants similar to the R9A(rep3) mutant were constructed such that Arg9 of each STPR repeat was individually replaced with Ala to generate three additional mutants: R9A(rep1), R9A(rep2), and R9A(rep4). We also generated and analyzed four C‐terminal truncation mutants and two N‐terminal truncation mutants: (a) ΔCtail, with a truncated C‐terminal tail; (b) ΔC‐rep4, with a truncated C‐tail and fourth repeat of the STPR domain; (c) ΔC‐STPR, with a truncated C‐tail, and all repeats of the STPR domain; (d) ΔC‐HBR, truncated from the C‐tail to the hyper basic region (HBR); (e) ΔN‐HBR, with a truncated N‐terminal region from the N terminus to HBR; and (f) ΔN‐AR, with a truncated from the N terminus to the acidic region (AR) (Fig. 9).

The mobility of each FMBP‐1 R9A mutant was observed in HeLa cells. Each R9A mutant localized in the nuclei, just as the R9A(rep3) mutant had in the PSG cells (Fig. 4D). Of the four R9A mutants, three: R9A(rep2), R9A(rep3), and R9A(rep4), did not show photobleaching (Fig. 7B). The position and shape of the autocorrelation curves of the R9A(rep3) mutant in HeLa cells were similar to those of the R9A(rep3) mutant in PSG cells (Fig. 6). The two‐component diffusion model was best for the curve fitting of the autocorrelation curves for these three R9A mutants (Fig. 5). The diffusion movements of the R9A(rep3) mutant in HeLa cells also closely reproduced those of the R9A(rep3) mutant in PSG cells. Additionally, the R9A(rep2) and R9A(rep4) mutants each showed results similar to those of the R9A(rep3) mutant. In contrast, the fluorescence intensity of the R9A(rep1) mutant decayed slightly in the first several seconds of each measurement, and the FCS data were best accounted for by the three‐component diffusion model (Fig. 5 and Table S4). Such differences among the R9A mutants were similar to those of previously published *in vitro* findings with respect to their DNA interactions 12. Thus, these findings suggested that each repeat of the STPR domain does not contribute equally to the DNA‐EGFP‐FMBP‐1 interactions and that the repeats might have differing roles.

**Figure 10:**
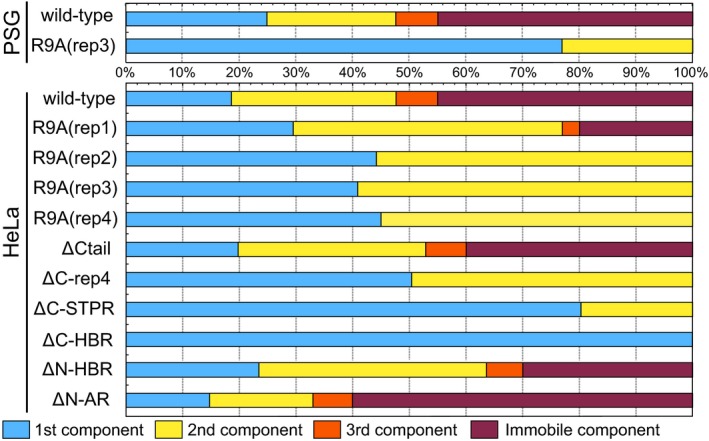
Component ratios among diffusion movements of EGFP‐FMBP‐1 mutants. The 100% stacked bar chart of average component ratios for each diffusion movement. This chart includes the immobile component ratio, which represents the decay rate of fluorescence intensity in photobleaching. The other three components (1st, 2nd, and 3rd) were distributed within the remaining component ratio, according to the component distribution *F*_i_ (*i* = 1, 2, 3) of diffusion parameters (obtained by fluorescence correlation spectroscopy (FCS) analyses, shown in Table 1 and 2).

The ΔCtail, ΔC‐rep4, ΔN‐HBR, and ΔN‐AR mutants each showed nuclear localization (Fig. 4E,F,I,J, respectively). Deletion of the entire STPR domain (ΔC‐STPR) resulted in reduced nuclear localization (Fig. 4G), and further deletion of HBR (ΔC‐HBR) eliminated nuclear localization completely (Fig. 4H). The ΔC‐rep4, ΔC‐STPR, and ΔC‐HBR mutants did not show photobleaching (Fig. 7B). In contrast, the decay of fluorescence intensity of three mutants: ΔCtail, ΔN‐HBR, and ΔN‐AR, was observed at an early stage of each FCS measurement, as was that of wild‐type FMBP‐1. For these photobleaching truncation mutants, the fluorescence fluctuation data from the observable photobleaching time period were removed from the ensemble average that was used for calculation of the autocorrelation curves. For curve fitting of the autocorrelation curves of the truncation mutants, the three‐component diffusion model was appropriate for each of the three mutants (Fig. 5). However, each of the other mutants: ΔC‐rep4, ΔC‐STPR, and ΔC‐HBR, did not show the 3rd component. Furthermore, the ΔC‐HBR mutant lost even the 2nd component. These diffusion parameters are shown in Table 2. The diffusion time of the 1st and 2nd components of each truncation mutant was within the same range as those for the wild‐type FMBP‐1. The component ratios between the immobile component and the three diffusion components of the respective FMBP‐1 mutants are summarized in Fig. 10. Notably, the ratio of the 2nd component for the mutant lacking the entire STPR (ΔC‐STPR) was decreased. Further deletion of HBR (ΔC‐HBR) clearly abolished the 2nd component. On the other hand, the ΔN‐HBR mutant, which had only the STPR domain and the C‐tail region, showed the 2nd component. These results indicated that the HBR and STPR domains each contributed to a fraction of the 2nd component and that the degree of these contributions differed.

### Fluorescence recovery after photobleaching analyses of wild‐type and mutant EGFP‐FMBP‐1 in HeLa cells

**Figure 11:**
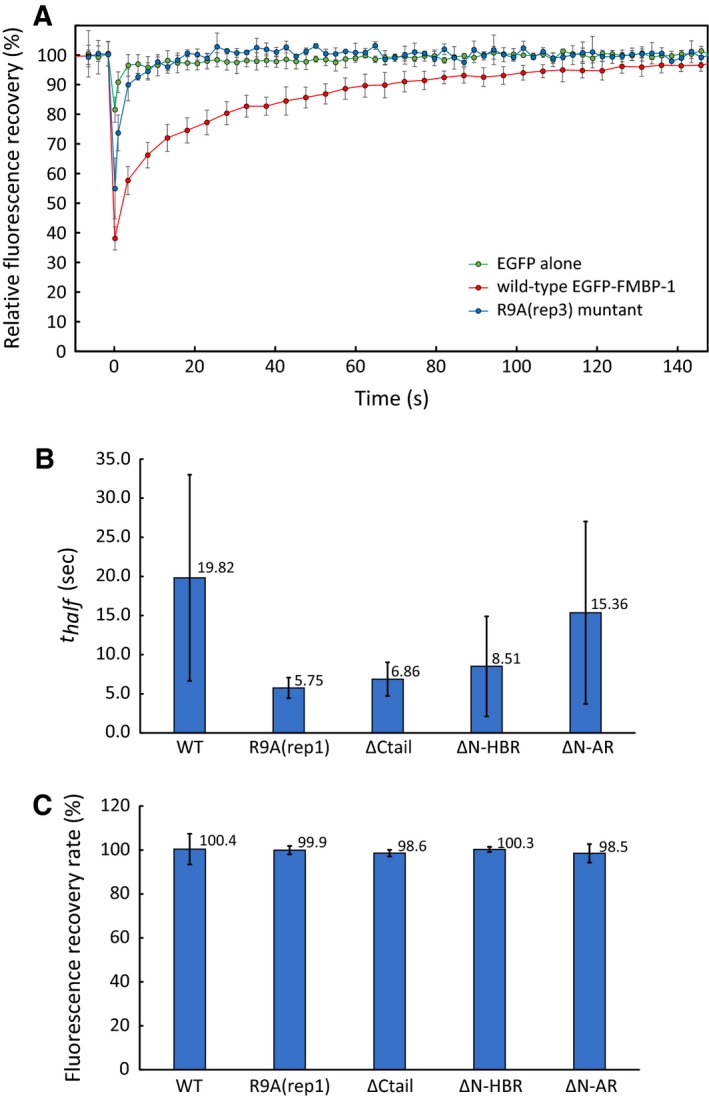
Fluorescence recovery after photobleaching (FRAP) of EGFP‐FMBP‐1 in HeLa cells. (A) Fluorescence recovery of wild‐type EGFP‐FMBP‐1 (red), R9A(rep3) mutant (blue) and EGFP alone (green) in HeLa cell nuclei. Averaged curves for wild‐type EGFP‐FMBP‐1 (*n* = 10), the R9A(rep3) mutant (*n* = 5), and EGFP alone (*n* = 5) are shown. The error bars show standard deviation. (B, C) Averaged *t*_half_ values of fluorescence recovery and fluorescence recovery rates, respectively, of wild‐type EGFP‐FMBP‐1 and mutants that show slow fluorescence recovery (*n* ≥ 10).

To characterize the immobile component of EGFP‐FMBP‐1 in more detail, we performed fluorescence recovery after photobleaching (FRAP) experiments in HeLa cells. The fluorescence recovery of wild‐type EGFP‐FMBP‐1 was clearly delayed compared to that of EGFP alone (Fig. 11A). The fluorescence recovery curves were fitted and interpreted using an exponential association model. The results of wild‐type EGFP‐FMBP‐1 showed that the fluorescence was completely recovered with *t*_half_ ≈20 s (Fig. 11B). For EGFP alone, we could not determine an accurate fluorescence recovery time in this experiment because the recovery was too rapid to analyze the recovery curve. In the nuclei of HeLa cells expressing the R9A(rep1), ΔCtail, ΔN‐HBR, and ΔN‐AR mutants, a slow fluorescence recovery (*t*_half_ ≈5–15 s) was also observed (Fig. 11B). In contrast, for HeLa cells expressing other mutants, the fluorescence recovery times could not be determined for the same reason as for EGFP alone. The fluorescence recovery rate of each EGFP‐FMBP‐1 mutant that showed slow fluorescence recovery also approached 100% (Fig. 11C). Notably, the EGFP‐FMBP‐1 mutants that showed slow fluorescence recovery in the FRAP experiments completely corresponded to the mutants exhibiting photobleaching during FCS analyses.

## Discussion

Our results indicated that STPR and HBR acted cooperatively to function as a nuclear localization signal (Fig. 4). Previous findings on human ZNF821, which contains a STPR motif in the C‐terminal tail, also indicated that STPR contributes to nuclear localization 23. Thus, the nuclear localization function of the STPR motif might be widely conserved among STPR‐containing proteins.

**Figure 12:**
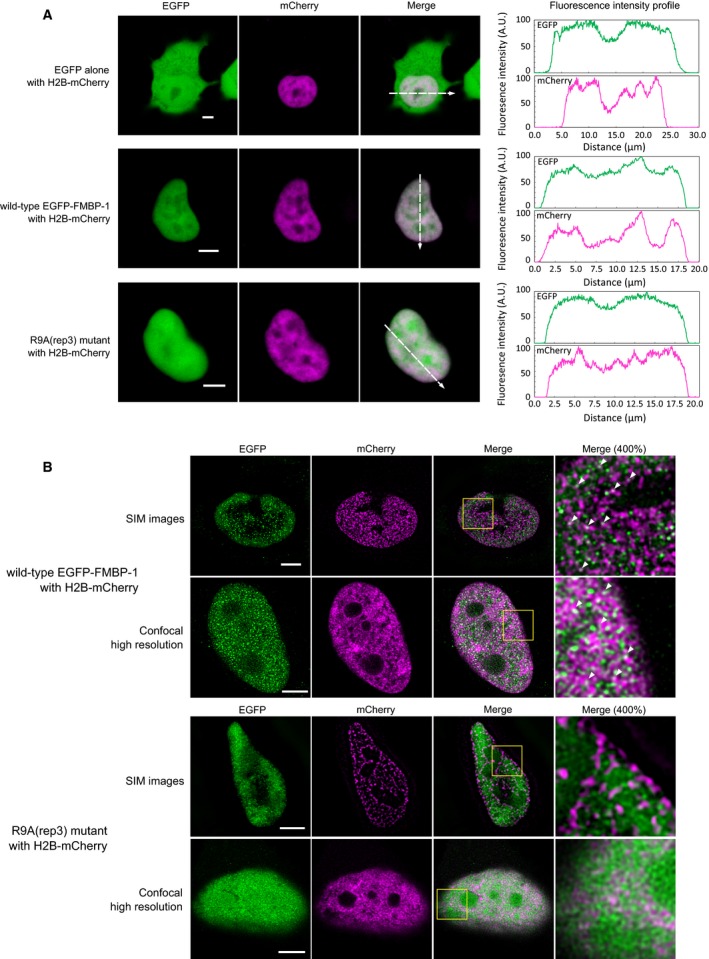
Colocalization of wild‐type EGFP‐FMBP‐1 and chromatin in HeLa cells. (A) Confocal images of HeLa cells in which EGFP, wild‐type EGFP‐FMBP‐1, or the R9A(rep3) mutant were coexpressed with histone H2B‐mCherry. The images were taken with a Carl Zeiss LSM510 microscope (EGFP fluorescence, Ex. 488 nm, Em. 505–530 nm; mCherry fluorescence, Ex. 594 nm, Em. LP650 nm). The scale bars represent 5 μm. The fluorescence intensity profile across the nucleus (shown as a white broken arrow) of each image is also shown for each fluorophore (right). (B) structured illumination microscopy (SIM) images and high‐resolution confocal images of HeLa cells in which wild‐type EGFP‐FMBP‐1, or the R9A(rep3) mutant were coexpressed with histone H2B‐mCherry. Those proteins were immunofluorescence stained by AlexaFluor 488 (for EGFP‐FMBP‐1), or AlexaFluor 594 (for H2B‐mCherry). The images were taken with a Nikon N‐SIM or Leica TCS SP8 microscope (AlexaFluor 488 fluorescence, Ex. 488 nm, Em. 504–595 nm; AlexaFluor 594 fluorescence, Ex. 595 nm, Em. 603–653 nm). The scale bars represent 5 μm. Expanded image of yellow frame region in each merge image is shown on the right. Arrow heads in expanded images indicate the colocalization spots of EGFP‐FMBP‐1 and H2B‐mCherry.

Although FCS analyses were carried out under low laser intensity, the EGFP‐tagged wild‐type FMBP‐1 showed observable photobleaching in both PSG and HeLa cells (Figs 2A and 7A). The photobleaching was also confirmed and analyzed in detail in HeLa cells using FRAP (Fig. 11). Previous studies suggest that FMBP‐1 binds to a specific recognition site consisting of a 9 bp sequence in the vicinity of the gene encoding the fibroin H chain, where it cooperates with other TFs to promote transcription 11. However, the length of this recognition sequence is very short in comparison with the genome size of *B. mori* (4.32 × 10^8^ bp, as the DNA content in a PSG cell polyploid nucleus becomes a thousand times larger than the original) and of humans (3.15 × 10^9^ bp). Thus, a rough estimation based on the expected frequency of this consensus sequence (every 8192 bp in the *B. mori* genome) indicated that multiple FMBP‐1 recognition sequences should be present throughout chromosomal DNA in both PSG and HeLa cells. Furthermore, previous electron microscopy findings showed that there are many euchromatic regions that can interact with TFs in the PSG cell nuclei of fifth instar larvae 2. Based on these estimations, we proposed that the photobleaching observed in both PSG and HeLa cells nuclei reflected the stable binding of FMBP‐1 to its recognition sequence throughout chromosomal DNA (Figs 2A and 7A). The disappearance of photobleaching caused by deletion or point mutation of the STPR domain suggested that STPR acted as the DNA‐binding domain of FMBP‐1 *in vivo*, as had been determined using previous *in vitro* assays (Figs 2B and 7B). To support the proposed interaction of EGFP‐FMBP‐1 with chromatin in HeLa cells, colocalization of EGFP‐FMBP‐1 and mCherry‐tagged histone H2B protein (H2B‐mCherry) was observed using confocal imaging of HeLa cells coexpressing wild‐type EGFP‐FMBP‐1 and H2B‐mCherry, wherein the distributions of both proteins overlapped (Fig. 12A). Super‐resolution imaging with structured illumination microscopy (SIM) and high‐resolution confocal imaging were also performed for detailed observation of the colocalization and to provide evidence of direct interaction, respectively. In the super‐resolution imaging of HeLa cells expressing wild‐type EGFP‐FMBP‐1, colocalization of EGFP fluorescence with H2B‐mCherry was clearly detected (Fig. 12B). In contrast, the EGFP fluorescence of the R9A(rep3) mutants did not completely colocalize with H2B‐mCherry in HeLa cell nuclei. Together, these results confirm the physical and localized interaction of wild‐type EGFP‐FMBP‐1 with chromatin. Thus, the photobleaching observed in the FCS analyses can likely be attributed to the stable binding of FMBP‐1 to chromatin. FRAP analysis of the stability of this interaction in HeLa cells (Fig. 11) determined that the *t*_half_ of the binding of wild‐type EGFP‐FMBP‐1 to chromatin was 20 s.

**Figure 13:**
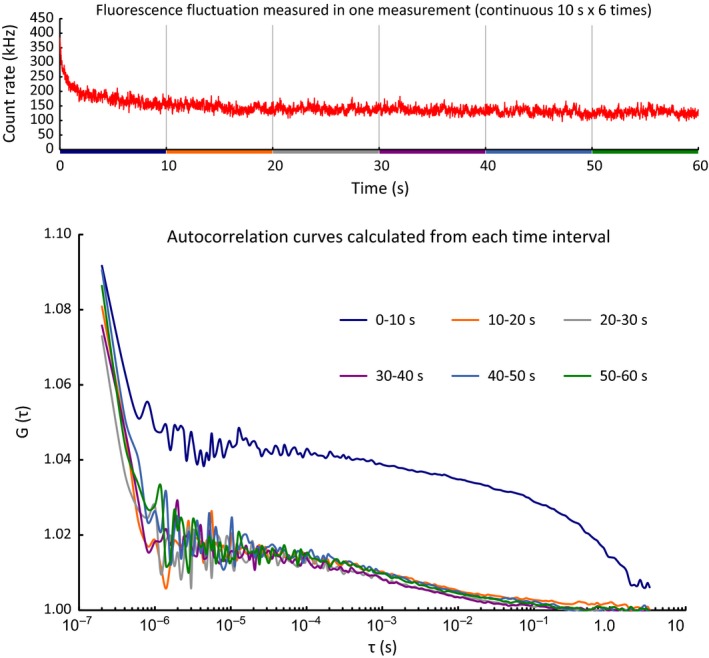
Autocorrelation curves derived from each 10‐s time interval of fluorescence fluctuation in a single fluorescence correlation spectroscopy (FCS) measurement. Each autocorrelation curve was calculated from each 10‐s time interval (0–10, 10–20, 20–30, 30–40, 40–50 and 50–60 s) of fluorescence fluctuation data (sequential 10 s × 6 times record) in a single measurement of wild‐type FMBP‐1 in posterior silk gland (PSG) cell. In the FCS analysis, first two time intervals were removed from the ensemble average of autocorrelation curve for curve fitting to determine the diffusion parameters.

In the FCS analyses, FMBP‐1 showed three distinct diffusion components in both PSG and HeLa cells based on curve fitting of the ACFs. Although the photobleaching period (the first 20 s of each measurement in PSG cells and the first 10 s in HeLa cells) was removed from the fluorescence fluctuation before curve fitting to determine the number of diffusion components (Fig. 3), the presence of 3rd component movement was coincident with the presence of photobleaching (Fig. 10). To check the possibility that the 3rd component was a remnant of photobleaching, we compared the autocorrelation curves that were derived from each 10‐s time interval of fluorescence fluctuation in each FCS measurement (consisting of six sequential 10 s of record data) (Fig. 13). The positions and shapes of the autocorrelation curves derived from the first 0–10 s of fluorescence fluctuation after the measurement start were clearly different from those of the autocorrelation curves derived from other time intervals. On the other hand, the autocorrelation curves derived from the five time intervals that represented later periods during data collection resembled each other. In addition, we determined the diffusion parameters by curve fittings of the autocorrelation curves derived from 20 to 30 or separately from 50 to 60 s of fluorescence fluctuations (Table S5). Generally, photobleaching can be accounted for by an exponential model 24. Thus, the influence of photobleaching would be expected to be weakest in the last 10‐s time interval. In our results, the component ratios and diffusion times of the 3rd diffusion component derived from 50 to 60 s did not decrease from those of the 20 to 30 s data, indicating that the 3rd component was not a remnant of photobleaching, but rather an independent component.

Because TFs generally bind to or interact with various molecules, one TF might show multiple distinct diffusion movements. Previous observations of TFs using FCS or FRAP have shown kinetic changes of the same TF via dimerization and ligand binding 18, 25, 26. Here, we found that the diffusion times of the 1st, 2nd, and 3rd component of each FMBP‐1 mutant were similar to those of the wild‐type FMBP‐1: τ_1_ = few hundred microseconds, τ_2_ = a few milliseconds, and τ_3_ = several tens of milliseconds or more, respectively (Tables 1 and 2); nevertheless, the component ratios clearly differed among the FMBP‐1 variants (Fig. 10). We therefore propose that these three components might reflect FMBP‐1 status during three different classes of movements and molecular interactions.

Based on the diffusion times measured via FCS, we assessed the apparent masses of each diffusing component. These calculations were based on the assumption that each diffusion component had a spherical shape, and previous work has stated that these calculations allow estimation of the order of the mass of the respective components 18. The apparent molecular masses of the diffusion components of FMBP‐1 measured in the PSG cells were calculated by comparing their diffusion times with that of EGFP alone (27 kDa). The apparent mass for the 1st component was approximately 58 kDa, nearly that of real monomeric EGFP‐FMBP‐1, 51.2 kDa. However, the apparent masses for both the 2nd component and the 3rd component were substantially larger than that of actual monomeric FMBP‐1 (approximately 50 000 kDa = 50 MDa and 1 300 000 000 kDa = 1.3 TDa, respectively). The ratio between the apparent molecular masses of the 2nd component and the real monomeric wild‐type FMBP‐1 molecule was approximately 1000‐fold. The apparent mass was also much larger than that of the preinitiation complex in transcription, which is approximately 3 MDa, or that of a mammalian ribosome, which is the largest intracellular organelle‐like complex and has a molecular mass of 4.6 MDa. Furthermore, for the 3rd component, the ratio between the apparent molecular masses and that of the actual monomeric wild‐type FMBP‐1 molecule was as high as 2.5 × 10^7^‐fold. It is unlikely that such a large diffusing protein complex exists in PSG cells. Thus, it is reasonable to interpret this slow diffusion not as representing the formation of extremely large molecular complexes, but as steric hindrance or transient interaction with another large and immobile structure in the cell nucleus. Actually, the delay of apparent diffusion times because of interactions with immobile structures has been proposed based on a previous simulation study involving FCS 27. Here, we did not observe any localization of EGFP‐FMBP‐1 on nuclear membranes or nucleoli in the confocal images; subsequently, those two nuclear regions were intentionally avoided during all FCS measurements (Fig. 4K,L). We also did not observe any aggregation or speckle formation in the nucleoplasm (Figs 1E and 4C). On the other hand, colocalization of the wild‐type EGFP‐FMBP‐1 with chromatin was confirmed by coexpression with H2B‐mCherry (Fig. 12). Thus, a probable candidate for such a large immobile structure that could interact with FMBP‐1 in the nucleus is chromosomal DNA. In short, these results strongly indicated that the slow diffusion movements (both the 2nd and 3rd components) of FMBP‐1 also reflected interactions, albeit transient, between FMBP‐1 and chromosomal DNA.

Recent studies of the interaction dynamics between DNA and TFs or DNA repair proteins using single‐molecule detection or molecular dynamics simulation have indicated that very short‐term events occur during protein–DNA interactions; such events are called ‘facilitated diffusion’ and include DNA sliding, DNA hopping, and DNA intersegment transfers. Importantly, facilitated diffusion has been suggested to cause proteins to reach a recognition sequence on DNA faster than would free, three‐dimensional diffusion 28, 29, 30, 31, 32, 33, 34, 35, 36. Notably, the diffusion coefficient of the 3rd component of wild‐type FMBP‐1 in the PSG cells in this study, *D* = 0.19 ± 0.20 μm^2^·s^−1^ (range 0.021–0.812 μm^2^·s^−1^) was comparable to that of DNA sliding as determined via single‐molecule detection (0.01–0.5 μm^2^·s^−1^) 31, 33, 34, 36. Additionally, the presence of 3rd component movement was coincident with the presence of photobleaching, which was considered to represent stable DNA binding with chromosomal DNA for all mutants (Fig. 10). Therefore, this result could be considered to support a model wherein the 3rd component movement represents facilitated diffusion that would trigger stable DNA binding. Previous findings have suggested that the DNA‐binding domains of TFs or DNA repair proteins might enter the groove of a DNA helix during the DNA sliding state; conformational changes in 3D structure are important for such interactions 32, 35. Findings from previous *in vitro* DNA‐binding experiments involving STPR domains indicate that a conformational change into the α‐helical‐rich structure is important for stable DNA binding and that the R9A mutation abolishes this conformational change 13. Thus, this conformational change might play an important role during 3rd component movements of the STPR as well as for stable DNA binding.

On the other hand, the diffusion coefficient of the 2nd component (*D* = 3.62 ± 2.07 μm^2^·s^−1^) was 19‐fold larger than that of the reported DNA sliding motion 31, 33, 34, 36. DNA hopping, which is another type of facilitated diffusion, is also reportedly more rapid than is DNA sliding 34, 35, 36. Here, we focused on the changes in the component ratios caused by mutation. In this study, every mutant that retained the STPR domain or HBR retained the 2nd component (Fig. 10). The isoelectric points calculated based on the amino acid sequences of HBR (pI = 12.5) or STPR (pI = 11.4) are very high, thus these positively charged regions could transiently adsorb onto the negatively charged DNA because of the electrostatic interactions between them. In fact, previous findings indicate that DNA hopping results from such electrostatic interactions and that the diffusion movement becomes faster with increasing salt concentrations 32, 35. It is likely that the 2nd component movements of FMBP‐1 therefore reflected the transient trapping of FMBP‐1 on chromosomal DNA (like DNA hopping) via electrostatic interactions in which both the STPR domain and HBR play important roles.

This study represents the first demonstration of FCS analyses in the silkworm silk gland *in vivo*. From this, we detected three types of interactions between EGFP‐FMBP‐1 and DNA using *in vivo* FCS and mathematical analyses involving a three‐component diffusion model. We note, however, that because of the complicated environment in living cells, the fitting analyses of FCS data often include some indeterminacy. Thus, the interpretation of molecular movements in living cells using only temporal autocorrelation analysis is not straightforward. In this study, the results of systematic mutation analyses support the interpretation suggested by FCS in living cells. Other fluorescence microscopic techniques were also performed to clarify the interaction target. Based on the results, we propose that the 1st, 2nd, 3rd, and immobile (photobleaching) components of the FCS analyses reflected (a) a free diffusion state of monomeric EGFP‐FMBP‐1 in cell nuclei, (b) a transient DNA interaction by electrostatic interaction on chromosomal DNA, (c) another transient DNA interaction that is strongly correlated with stable DNA binding, and (d) the final stable binding state, respectively. These components might correspond to each step that FMBP‐1 takes in the recognition of its DNA‐binding sequence. In particular, as the polyploid nuclei of the PSG cells hold extremely large DNA contents, the two types of transient interactions observed in this study might play important roles, allowing FMBP‐1 proteins to reach their final recognition sequence sites on the chromosomal DNA for the efficient expression of the fibroin gene.

## Materials and methods

### Plasmid constructs

A cDNA encoding FMBP‐1 was PCR amplified and then subcloned into the pEGFP‐C1 vector (Clontech, Palo Alto, CA, USA) downstream of the EGFP‐coding region; *Hin*dIII and *Kpn*I were used to subclone the cDNA. Mutants consisting of R9A(rep1) (substitution of alanine for the ninth arginine of repeat 1 [R107] of the STPR domain of FMBP‐1), R9A(rep2) (substitution of alanine for the ninth arginine of repeat 2 [R130]), R9A(rep3) (substitution of alanine for the ninth arginine of repeat 3 [R153]), R9A(rep4) (substitution of alanine for the ninth arginine of repeat 4 [R176]), ΔN‐HBR (a deletion extending from M1 to G95 of FMBP‐1), ΔN‐AR (a deletion extending from M1 to L82), ΔCtail (a deletion extending from K191 to T218), ΔC‐rep4 (a deletion extending from E168 to T218), ΔC‐STPR (a deletion extending from E99 to T218), and ΔC‐HBR (a deletion extending from K86 to T218) were each created using the QuikChange Site‐Directed Mutagenesis Kit (Stratagene, La Jolla, CA, USA) according to the manufacturer's instructions. The integrity of each plasmid was confirmed via DNA sequencing.

### Transient expression in silk gland cells

The silk glands from fifth instar, day 0 *B. mori* larvae were dissected. Then, the Helios Gene Gun system (Bio‐Rad, Hercules, CA, USA) was used as previously described 37 to introduce tungsten particles coated with plasmid DNA into silk gland tissues incubated in Petri dishes. Each silk gland was transplanted into another fifth instar on day 0; the transplants were incubated at 25 °C for 17 h. After incubation, the transplanted silk glands were removed from the host larvae. Then, the PSG tissues were separated from each transplanted silk gland and mounted on a slide glass immediately before laser scanning microscopy (LSM) imaging and FCS measurement. During these procedures, PSG tissues were maintained in phosphate‐buffered saline (PBS). For transient expression in both PSG and HeLa cells, the CMV promoter, which is encoded in the mammalian expression vector pEGFP‐C1, was used to drive EGFP‐FMBP‐1 expression. Expression levels of EGFP‐FMBP‐1 were high enough to make FCS measurements.

### Transient expression in HeLa cells

HeLa cells were plated in eight‐well Lab‐Tek Chambered coverslips (Nalge Nunc International, Rochester, NY, USA) and incubated in Dulbecco's modified Eagle's medium (DMEM; Sigma‐Aldrich, St. Louis, MO, USA) supplemented with 10% fetal bovine serum, 100 units·mL^−1^ penicillin, and 100 μg·mL^−1^ streptomycin in a 5% CO_2_ humidified atmosphere at 37 °C for 24 h. Transfection was conducted using Optifect transfection reagent (Invitrogen, Carlsbad, CA, USA) according to the manufacturer's instructions. Cells were then grown in DMEM supplemented with 10% fetal bovine serum for 24 h in a 5% CO_2_ humidified atmosphere. During LSM imaging and FCS measurement, HeLa cells were maintained in Opti‐MEM reduced‐serum medium (Invitrogen).

### LSM imaging and FCS measurement

Both LSM imaging and FCS measurements were performed using an LSM510 inverted confocal laser scanning microscope (Carl Zeiss, Jena, Germany), which comprised a CW Ar+ laser, a water immersion objective (C‐Apochromat, 40×, 1.2 NA; Carl Zeiss), and a ConfoCor 3 (Carl Zeiss). For LSM imaging of PSG cells, the cells were stained with 25 μg·mL^−1^ Hoechst 33342 (Invitrogen) to visualize the nuclei. The Hoechst dye was excited by a 405‐nm diode laser, and the emission signal was detected through a 420–480 nm band‐pass filter. EGFP was excited by a 488 nm laser, and the emission signal was detected through a 505–540 nm band‐pass filter.

Each FCS measurement was performed in the nucleus of an individual cell, avoiding the nuclear membranes and nucleoli. The output level of the excitation laser was kept at constant intensity during each measurement, at 112.5 μW or 225 μW for PSG or HeLa cells, respectively. The actual values of the laser intensity on the stage of the microscope were approximately 2.5 μW during each measurement for both cell types. The sequential illumination periods during the measurement of fluorescence intensity were 15.0 μs each. The data were processed in essentially the same manner as described previously 23, except that the measurement time (60 s, consisting of six consecutive measurements of 10 s each) differed. For quantitative analyses, the fluorescence ACF *G(*τ*)* was calculated as follows:

(1) $$G\left(\tau \right)=\frac{\left\langle I\left(t\right)I\left(t+\tau \right)\right\rangle }{\langle I{\left(t\right)}^{2}\rangle },$$

where τ represents the time delay, *I* is the fluorescence intensity, and the brackets denote the ensemble average. The binning period for extracting the ACF was 0.2 μs. To calculate the decay rate of fluorescence intensity for samples that showed photobleaching, the fluorescence fluctuations were fitted by the following equation:

(2) $$y=A*\exp\left(-t*k\right)+y_{0},$$

where *A* is the height of photobleaching, *k* is the rate constant of photobleaching, and *y*_0_ is the steady‐state level of fluorescence fluctuation. Then, the fluorescence fluctuation data in the photobleaching time period (first 20 s or 10 s for PSG cells or HeLa cells, respectively) were removed from the temporal and ensemble average to extract the ACF.

*G*(τ) was fitted with lsm‐fcs software (Carl Zeiss) by multicomponent diffusion models that are described as follows:

(3) $$G\left(\tau \right)=\frac{1}{N}\left[\sum_{i=1}^{M}\frac{F_{i}}{{\left(1+(t/\tau_{i}\right)}^{\alpha })\sqrt{1+1/S^{2}{\left(t/\tau_{i}\right)}^{\alpha }}}\right]+1,$$

where *M* is the number of fluorescent components and *N* is the average number of fluorescent molecules in the detection volume. *F*_*i*_ and τ_*i*_ are the component ratio and translational diffusion time of the *i‐*th fluorescent component, respectively. *S* is the structural parameter of the instrumental set up *S* = *z*/ω, where ω and *z* are the e^−2^ beam radius in the lateral and the axial directions of focus, respectively. The exponent α accounts for the mechanism of diffusion; α = 1 for free (Brownian) diffusion and α < 1 for obstructed (anomalous) diffusion. The fitting model did not include any expression for the triplet‐state relaxation of EGFP, as triplet‐state relaxation has a much longer lifetime than that of general fluorescence. Therefore, triplet‐state relaxation often causes miscalculation of the diffusion parameters. If the influence of triplet‐state relaxation substantially affected the extracted functions, we could use fitting models that included an expression for this; however, when we included an expression for the triplet‐state term in the fitting model, it had very little influence on the results (Table S6). In this study, the fitting ranges for ACFs were set from τ ≈ 50 μs to 3.4 s in order to avoid large variation in ACFs at early time points. Triplet‐state relaxation has been reported to influence the extracted functions mainly at the early ACF (at τ ≈ few μs); generally, the influence of triplet‐state relaxation decreases exponentially. Thus, the influence of triplet‐state relaxation should be weak in the fitting range (τ ≈ 50 μs to 3.4 s) for our FCS analyses. Therefore, we used a fitting model that did not include an expression for the triplet‐state term in this study.

In the case of ACFs with correlations that were too small (due to high fluorescence intensity caused by a high concentration of EGFP‐tagged protein) to fit a curve normally, the function was removed from the dataset. As a result, the average of the concentration of EGFP‐FMBP‐1 in this FCS measurement was approximately 400 nm (the range was ≈ 100–1000 nm).

The diffusion coefficient *D*_i_ (*i* = 1, 2, 3) of a fluorescent species could then be assessed as follows:

(4) $$D_{i}=\omega^{2}/\left(4\tau_{i}\right),$$

where ω at 488 nm excitation was estimated from the diffusion coefficient of rhodamine 6G (R6G) 38.

The apparent molecular masses of EGFP‐FMBP‐1 monomers or EGFP‐FMBP‐1‐containing complexes (*Mw*_*i*_), assuming a spherical shape for the diffusing species, were assessed from the diffusion times by using the following mathematical expression based on the Stokes–Einstein equation as follows:

(5) $$Mw_{i}=27{\left(\frac{\tau_{i}}{\tau_{\mathrm{EGFP}}}\right)}^{3}$$

The molecular mass of EGFP is 27 kDa. τ_EGFP_ is the diffusion time of EGFP alone when measured under the same experimental conditions as EGFP‐FMBP‐1.

The statistical analysis used to compare the averages of the best fit parameters was carried out with unpaired *t*‐tests using microsoft excel 2013 software (Microsoft, Redmond, WA, USA). To examine the appropriate number of components for the fitting model, a comparison of different fitting models was carried out using AIC and with *F*‐tests, also using microsoft excel 2013 software, where:

(6) $$\text{AIC}=N\cdot \ln\left(\frac{\chi^{2}}{N}\right)+2K+\frac{2K(K+1)}{N-K-1}$$

*N* is the number of data points used for each curve fitting, and *K* is the number of parameters. More precisely, the formula showed a corrected AIC that was corrected for the influence of sample size:

(7) $$F=\frac{\left(\chi_{1}^{2}-\chi_{2}^{2}\right)/\left(DF_{1}-DF_{2}\right)}{\chi_{2}^{2}/DF_{2}}$$

where χ^2^_1_ is the χ^2^ for the simpler model and χ^2^_2_ is the χ^2^ for the more complicated model. DF is the degree of freedom (N‐K‐1). *P*‐values for each F‐ratio were calculated using the ‘FDIST’ formula of Microsoft Excel 2013. The χ^2^ values for each fitting were calculated as follows:

(8) $$\chi^{2}=\sum_{i}{\left[\frac{y\left(x_{i}\right)-y_{i}}{\sigma_{i}}\right]}^{2}$$

The formula shows the difference between the fitted function *y*(*x*) and the experimental data *y*_*i*_ at points *x*_*i*_ weighted by σ_*i*_ as a sum over all data points *i*, where σ_*i*_ is the standard deviation for experimental point *i*.

For the statistical analysis, we referred to articles 18, 22, 39 and a manual for the online graphpad Software (http://www.graphpad.com/quickcalcs/AIC2.cfm).

### FRAP measurement

FRAP measurements were performed in essentially the same manner as described previously 40, 41. For the measurements, an LSM510 inverted confocal laser scanning microscope (Carl Zeiss) with same settings as for FCS measurements was used. A small area (approximately 3.0 μm diameter circle) was positioned in a region of the nucleus that did not contain the nucleolus, and bleached using 100% Ar^+^ laser power (7.5 mW) with 15 scans. Images were then collected using 2.0% laser power every 2.5 s for 3.0 min (for the samples showing fast fluorescence recovery) or every 5.0 s for 6 min (for those with slow fluorescence recovery). The relative fluorescence intensities in the bleached area were normalized using the average intensity of three images measured before bleaching. The normalized intensities were analyzed using a fitting equation for a single exponential association model.

### Confocal imaging for observation of colocalization

To observe the colocalization of EGFP‐FMBP‐1 and chromatin, we utilized a plasmid encoding mCherry‐tagged histone H2B protein (H2B‐mCherry), which was constructed by substitution of mCherry for GFP in the pBOS‐H2BGFP vector 42. The H2B‐mCherry plasmid DNA was transiently coexpressed with EGFP‐FMBP‐1 in HeLa cells. For confocal imaging, an LSM510 inverted confocal laser scanning microscope (Carl Zeiss) with same settings as those for FCS measurements was used. The mCherry was excited by a 594 nm diode laser, and the emission signal was detected through a 650 nm long‐pass filter.

### Immunofluorescence staining and super‐resolution fluorescence microscopy

Cells were cultivated on glass‐based dishes (0.14–0.18 mm) (#3970‐035, AGC Techno glass, Shizuoka, Japan), and then fixed with 4% paraformaldehyde buffered with Hepes‐KOH (pH 7.5) at 37 °C. The cells were washed in TBS and permeabilized in the presence of 0.2% (v/v) Triton X‐100 (Nacalai tesque, Kyoto, Japan). After blocking nonspecific binding activity in a blocking buffer containing 5% normal goat serum (DAKO, Glostrup, Denmark) and 20% Glycerol in PBS, the cells were incubated for 1 h at room temperature in blocking buffer supplemented with primary antibodies against EGFP (#GF200; Nacalai tesque) and DsRed (#632496; TaKaRa‐Clontech). The cells were then incubated with anti‐mouse IgG antibody conjugated with AlexaFluor 488 or anti‐rabbit IgG antibody conjugated with Alexa Fluor 594 (Thermo Fisher Scientific, Waltham, MA, USA) in blocking buffer for 1 h at room temperature. Cells stained on cover slips were mounted with ProLong Gold (Thermo Fisher Scientific). For super‐resolution SIM, images were captured on an N‐SIM system (Nikon, Tokyo, Japan) equipped with an Apo TIRF 100×/1.49 NA oil‐immersion objective (Nikon). Alexa Fluor 488 was excited at 488 nm, and Alexa Fluor 594 was excited at 561 nm. Image acquisition and SIM‐reconstruction were operated on an nis‐elements software platform (Nikon). For high‐resolution confocal microscopy, images were captured on a laser confocal microscope TCS SP8 (Leica, Wetzlar, Germany) equipped with an HC PL APO CS2 100×/1.40 OIL STED WHITE objective (Leica). AlexaFluor 488 and 594 was excited at 488 and 595 nm, respectively. Emitted fluorescence was sequentially detected using a photon‐counting HyD detector through a spectral separation module (504–595 nm for AlexaFluor 488 and 603–653 nm for AlexaFluor 594). A pinhole size was set at 0.60 airy units (91 μm). Acquired images were processed using a deconvolution algorithm on a huygens software platform (Scientific Volume Imaging, Hilversum, Netherlands).

## Author contributions

MT, HM, AK, ST, MKinjo, and TA planned experiments. MT, HM, SM, MKimoto, and AK performed experiments. MT, HM, and SM analyzed data. MKamiya, TK, ST, MD, KK, MKinjo, and TA contributed new reagents/analytic tools. MT, HM, MKinjo, and TA wrote the paper.

## Supporting information


**Table S1.** AIC test of each fitting model for wild‐type EGFP‐FMBP‐1 data measured in PSG cells.
**Table S2.** *F*‐test of the three‐component model against the two‐component model for wild‐type EGFP‐FMBP‐1 data in PSG cells.
**Table S3.** Diffusion parameters of wild‐type EGFP‐FMBP‐1 in PSG obtained by various diffusion models.
**Table S4.** Examination of the appropriateness of the interpretation with the three‐component model for the R9A(rep1) mutant in HeLa cells.
**Table S5.** Influence of photobleaching for diffusion parameters.
**Table S6.** Influence of triplet‐state relaxation for diffusion parameters.
