# Supplementary material for: In vivo fluorescence correlation spectroscopy analyses of FMBP‐1, a silkworm transcription factor
Source: FEBS Open Bio. 2016 Jan 27;6(2):106–25. doi: 10.1002/2211-5463.12026 (PMC4821344; doi:10.1002/2211-5463.12026)
Supplement: Supplementary file 1 — Table S1. AIC test of each fitting model for wild‐type EGFP‐FMBP‐1 data measured in PSG cells. Table S2. F‐test of the three‐component model against the two‐component model for wild‐type EGFP‐FMBP‐1 data in PSG cells. Table S3. Diffusion parameters of wild‐type EGFP‐FMBP‐1 in PSG obtained by various diffusion models. Table S4. Examination of the appropriateness of the interpretation with the three‐component model for the R9A(rep1) mutant in HeLa cells. Table S5. Influence of photobleaching for diffusion parameters. Table S6. Influence of triplet‐state relaxation for diffusion parameters. [file FEB4-6-106-s001.pdf]

In vivo fluorescence correlation spectroscopy analyses of FMBP-1, a silkworm transcription factor

Supporting Information

Motosuke Tsutsumi [a], Hideki Muto [a,b], Shohei Myoba [a], Mai Kimoto [c], Akira Kitamura [a], Masakatsu Kamiya [a], Takashi Kikukawa [a], Shigeharu Takiya [c], Makoto Demura [a], Keiichi Kawano [a,d], Masataka Kinjo [a,*], Tomoyasu Aizawa [a,*]

[a] Faculty of Advanced Life Science, Hokkaido University, N10, W8, Sapporo, Hokkaido 060-0810, Japan
[b] Biomedical Research Support Center, Nagasaki University School of Medicine, 1-12-4 Sakamoto, Nagasaki, Nagasaki 852-8523, Japan
[c] Faculty of Science, Hokkaido University, N10, W8, Sapporo, Hokkaido 060-0810, Japan
[d] Chitose Institute of Science and Technology, 758-65 Bibi, Chitose, Hokkaido 066-8655, Japan

Table S1. AIC test of each fitting model for wild-type EGFP-FMBP-1 data measured in PSG cells

Measurement	Number of data points	1 component, free-diffusion model		2 component, free-diffusion model		3 component, free-diffusion model	
		Chi²	AIC	Chi²	AIC	Chi²	AIC
1	134	97.3	-38.8	13.7	-297.2	13.1	**-299.3**
2	132	192.6	53.9	18.0	-254.4	15.0	**-274.2**
3	135	195.2	53.9	25.4	-217.0	8.4	**-362.7**
4	134	159.2	27.2	23.1	-227.2	10.6	**-327.9**
5	126	415.0	154.3	37.0	-146.0	32.6	**-157.8**
6	132	223.9	73.8	32.5	-176.8	20.8	**-231.0**
7	142	379.9	143.8	45.6	-153.1	35.6	**-183.7**
8	132	443.4	164.0	35.2	-166.1	32.7	**-171.4**
9	126	272.2	101.1	54.2	-98.0	51.7	**-99.5**
10	131	743.4	231.5	39.8	-147.7	27.5	**-191.9**
11	127	170.3	41.4	17.5	-243.0	11.2	**-295.2**
12	140	93.4	-52.5	16.0	-295.7	15.5	-295.4
13	130	149.7	22.4	29.7	-183.8	19.8	**-232.0**
14	134	410.2	154.0	22.8	-228.8	19.4	**-246.4**
15	133	295.7	110.4	30.4	-188.1	11.6	**-311.9**
16	129	652.7	213.2	33.2	-166.6	27.8	**-185.3**
17	119	1331.7	291.5	42.9	-113.1	39.2	**-119.3**
18	118	164.9	43.6	29.9	-153.5	25.8	**-166.7**
19	130	213.6	68.6	28.7	-188.3	28.1	-186.3
20	134	183.7	46.3	18.7	-255.4	13.0	**-300.0**
21	136	108.7	-26.4	16.5	-278.9	12.2	**-315.3**
22	127	212.9	69.7	32.4	-165.0	30.1	**-170.1**
23	129	577.3	197.4	29.0	-184.1	28.0	**-184.4**
24	131	765.7	235.4	58.5	-97.2	53.5	**-104.6**
25	133	169.6	36.4	17.7	-259.6	10.0	**-331.4**
26	132	380.4	143.8	29.9	-187.8	13.8	**-285.6**
27	130	308.0	116.2	19.2	-240.5	15.9	**-260.7**
28	128	166.2	37.5	19.9	-229.7	13.3	**-277.0**
29	124	486.5	173.6	23.9	-195.8	20.7	**-209.4**

The AIC values were calculated from the χ^2 of curve fitting for wild-type FMBP-1 in PSG cells (total 29 fitting). Each AIC value was generated using the formula *(6)* shown in *Materials and Methods*. AIC represents the appropriateness of the fitting model used in curve fitting. The appropriateness is judged by the value; lower values indicate a more probable fitting model. AIC values in bold type in the three-component, free-diffusion model column are the lowest among the three different models.

Table S2. *F*-test of the three-component, free-diffusion model fitting against the two-component, free-diffusion model for wild-type EGFP-FMBP-1 data in PSG cells

Measurement	*F*-value	*P*-value
1	3.15	**<0.05**
2	12.59	**<0.005**
3	130.50	**<0.005**
4	75.61	**<0.005**
5	8.19	**<0.005**
6	34.92	**<0.005**
7	18.87	**<0.005**
8	4.74	**<0.05**
9	2.80	0.06
10	27.83	**<0.005**
11	33.66	**<0.005**
12	1.93	0.15
13	30.61	**<0.005**
14	11.29	**<0.05**
15	102.13	**<0.005**
16	11.92	**<0.005**
17	5.22	**<0.05**
18	8.96	**<0.005**
19	1.16	0.32
20	28.01	**<0.005**
21	22.54	**<0.005**
22	4.65	**<0.05**
23	2.22	0.11
24	5.82	**<0.05**
25	48.68	**<0.005**
26	73.07	**<0.005**
27	12.79	**<0.005**
28	30.08	**<0.005**
29	9.13	**<0.005**

Using Microsoft Excel 2013 software, *F*-tests were conducted to compare the χ^2 values of the three-component model with those of the two-component model for each of the 29 measurements from the wild-type EGFP-FMBP-1 measured in PSG cells. F-values were calculated by the formula *(7)* shown in *Materials and Methods*. *F*-values and the corresponding *P*-values or *P*-value upper bounds are listed for each measurement in the Table. Significant values (<0.05) are shown in bold.

Table S3. Diffusion parameters of wild-type EGFP-FMBP-1 in PSG obtained by various diffusion models

(a) Two-component, free-diffusion model

1st component		2nd component	
F_1 (%)	τ_1 (μs)	F_2 (%)	τ_2 (ms)
70.8 ± 7.1	678.7 ± 169.3	29.2 ± 7.1	22.03 ± 12.81

(b) One-component, anomalous-diffusion model

1st component (anomalous)	
τ_1 (μs)	α
1188.8 ± 462.6	0.63 ± 0.05

(c) Two-component, anomalous-diffusion model (one components is anomalous; the other is free)

1st component (anomalous)			2nd component (free)	
F_1 (%)	τ_1 (μs)	α	F_2 (%)	τ_2 (ms)
75.1 ± 19.6	3058.0 ± 7535.0	0.76 ± 0.12	24.9 ± 19.6	48.27 ± 89.96

(d) Two-component, anomalous-diffusion model (both components are anomalous)

1st component (anomalous)			2nd component (anomalous)		
F_1 (%)	τ_1 (μs)	α	F_2 (%)	τ_2 (ms)	α
71.4 ± 28.2	788.4 ± 446.7	0.86 ± 0.43	28.6 ± 28.2	1.28E+8 ± 6.76E+8	1.44 ± 1.23

(e) Three-component anomalous-diffusion model (all components are anomalous)

1st component (anomalous)			2nd component (anomalous)			3rd component (anomalous)		
F_1 (%)	τ_1 (μs)	α	F_2 (%)	τ_2 (ms)	α	F_3 (%)	τ_3 (ms)	α
44.9 ± 35.6	482.3 ± 403.9	33.95 ± 107.57	35.8 ± 30.2	5.82 ± 8.49	1.69 ± 1.15	19.3 ± 19.9	96.8 ± 159.5	5.65 ± 18.25

Note: α values are anomaly parameters; $\alpha = 1$ for free (Brownian) diffusion, $\alpha < 1$ for obstructed (anomalous) diffusion.

(f) Comparison of AIC values between the three-component, free-diffusion model and each anomalous-diffusion model.

	1-component, anomalous		2-component, anomalous (one of the pair is anomalous)		2-component, anomalous (both components are anomalous)		3-component, anomalous (all components are anomalous)		3-component, free
Measurement	Chi²	AIC	Chi²	AIC	Chi²	AIC	Chi²	AIC	AIC
1	16.5	-274.7	13.1	-301.4	12.8	-301.7	12.4	-301.7	-299.3
2	20.3	-241.1	15.2	-274.7	14.9	-275.2	12.6	-292.7	-274.2
3	10.5	-338.0	7.5	-380.5	7.5	-377.4	7.3	-377.2	-362.7
4	9.9	-343.1	10.1	-335.9	9.8	-337.6	9.0	-344.6	-327.9
5	39.4	-140.2	31.7	-163.2	21.9	-208.0	21.3	-206.8	-157.8
6	22.7	-226.0	19.1	-244.6	19.2	-242.1	17.3	-251.2	-231.0
7	37.7	-182.3	35.7	-185.8	33.6	-192.0	35.2	-180.8	-183.7
8	42.4	-143.6	32.4	-175.0	31.5	-176.7	30.1	-174.5	-171.4
9	54.2	-100.2	51.3	-102.7	48.4	-107.8	47.5	-105.7	-99.5
10	51.3	-116.6	28.2	-190.9	26.6	-196.0	26.2	-193.7	-191.9
11	14.4	-269.9	10.8	-302.9	10.8	-300.3	9.7	-309.5	-295.2
12	20.3	-264.4	18.6	-272.2	18.2	-272.7	17.8	-271.6	-295.4
13	20.9	-231.6	20.1	-232.0	19.7	-232.8	19.6	-228.5	-232.0
14	25.1	-218.5	18.3	-256.5	17.6	-259.6	16.4	-264.6	-246.4
15	25.1	-215.6	8.9	-349.3	8.8	-348.6	8.4	-350.4	-311.9
16	35.3	-161.1	28.6	-183.8	25.1	-198.3	27.3	-182.9	-185.3
17	65.7	-64.5	44.4	-106.9	36.1	-129.1	18.7	-202.7	-119.3
18	31.6	-149.4	26.0	-167.8	26.7	-162.6	23.4	-176.5	-166.7
19	45.7	-129.8	27.6	-190.9	23.5	-209.7	23.1	-207.5	-186.3
20	18.8	-257.1	13.1	-301.5	13.0	-300.0	10.4	-325.4	-300.0
21	10.8	-338.1	12.1	-318.3	11.3	-325.2	11.4	-320.6	-315.3
22	38.3	-146.1	30.2	-172.0	30.9	-166.9	29.0	-170.3	-170.1
23	53.7	-106.8	27.6	-188.6	23.1	-209.4	23.1	-204.8	-184.4
24	48.4	-124.1	44.2	-131.8	48.4	-117.8	48.3	-113.6	-104.6
25	9.9	-338.9	10.0	-333.8	9.7	-335.8	9.4	-335.4	-331.4
26	16.6	-267.8	14.6	-280.1	14.8	-276.6	13.1	-287.4	-285.6
27	18.7	-246.1	16.6	-257.4	14.6	-271.2	11.8	-295.0	-260.7
28	12.8	-288.3	11.4	-299.0	11.7	-293.8	10.4	-303.5	-277.0
29	19.0	-226.6	19.7	-217.3	19.0	-219.7	14.9	-257.8	-209.4

The AIC values calculated from the fitting residuals of wild-type FMBP-1 in PSG cells (total 29 fitting). This comparison of AIC values was processed in the same manner as described for Table S1. In more than half of the fitting samples, the one-component, anomalous-diffusion model was judged to be improbable compared with the three-component, free-diffusion model. The other two anomalous-diffusion models were judged to be relatively probable. However, the differences in AIC values between the two anomalous-diffusion models and those of the three-component free-diffusion model were not large for most fitting samples. For reference, the chi^2 and AIC values for the three-component, anomalous diffusion model are also displayed.

Table S4. Examination of the appropriateness of the interpretation with the three-component model for the R9A(rep1) mutant in HeLa cells

(a) Diffusion parameters of the R9A(rep1) mutant determined by using the two-component, free-diffusion model

1st component		2nd component	
F_1 (%)	τ_1 (μs)	F_2 (%)	τ_2 (ms)
63.7 ± 12.2	617.9 ± 174.4	36.3 ± 12.2	6.62 ± 3.44

In comparison with the diffusion parameters obtained by the three-component free-diffusion model (shown in Table 2), the component ratios and diffusion times of each component were clearly different. Also, dispersion of each parameter was increased.

(b) Comparison of fitting residuals of the R9A(rep1) mutant by the three-component model and the two-component model

Measurement	3-component model		2-component model	
	Chi²	AIC	Chi²	AIC
1	10.0	-352.8	22.2	-246.6
2	5.6	-395.9	12.1	-300.7
3	21.9	-207.6	24.8	-196.5
4	8.6	-358.6	12.0	-318.3
5	21.2	-223.4	29.6	-183.9
6	16.7	-253.7	27.0	-195.8
7	29.0	-200.1	36.6	-172.6
8	10.2	-314.9	11.6	-302.5

The AIC values calculated from the fitting residuals of R9A(rep1) mutants in HeLa cells (total 8 fitting). This comparison of AIC values was processed in the same manner as described in Table S1. In all eight fittings, the three-component free diffusion model was judged to be more probable than the two-component model.

Table S5. Influence of photobleaching for diffusion parameters

	1st component		2nd component		3rd component		n
	F_1 (%)	τ_1 (μs)	F_2 (%)	τ_2 (ms)	F_3 (%)	τ_3 (ms)	
wild-type FMBP-1 (20-30 sec)	41.5 ± 11.7	299.1 ± 77.4	43.5 ± 8.0	2.78 ± 1.61	14.9 ± 11.1	114.6 ± 75.7	16
wild-type FMBP-1 (50-60 sec)	37.5 ± 17.1	330.7 ± 63.5	43.6 ± 14.8	2.86 ± 1.96	18.9 ± 9.9	73.3 ± 51.9	19

These values are derived by curve fitting of the autocorrelation function (ACF) calculated from the third (20-30 sec) or last (50-60 sec) 10 seconds of continuous fluorescence fluctuation data (10 sec × 6 times) of wild-type FMBP-1 in PSG cells with the three-component, free-diffusion model. Each of 16 or 19 samples of all 29 measured samples could be fit. However, other samples could not be fit well, resulting in very few component ratios and large diffusion times for each component. It is impossible to consider the ratios and times are real values. Such difficulty of curve fitting would be caused by large variation of the ACF, calculated from shorter fluorescence fluctuation than normal curve fitting of ACF in this study (which was calculated from continuous 10 × 4 times measurement data).

Table S6. Influence of triplet-state relaxation for diffusion parameters

	1st component		2nd component		3rd component		
	F_1 (%)	τ_1 (μs)	F_2 (%)	τ_2 (ms)	F_3 (%)	τ_3 (ms)	*n*
wild-type FMBP-1	45.6 ± 13.7	347.4 ± 56.9	41.1 ± 7.1	3.37 ± 1.70	13.3 ± 9.6	102.1 ± 88.9	28

These values are derived by curve fitting of the autocorrelation function of wild-type FMBP-1 in PSG cells with the three-component diffusion model incorporating triplet-state relaxation as follows:

$$G(\tau) = \frac{1 + \frac{T}{1-T} e^{-t/\tau_T}}{N} \left[\sum_{i=1}^{M} \frac{F_i}{(1 + t/\tau_i)\sqrt{1 + t/(S^2 \tau_i)}} \right] + 1.$$

The triplet-state term was inserted in each fitting model that was used for the present study (shown in *Materials and Methods*). T and τ_T are the fractional population and decay time of the triplet state, respectively. We tested the fitting model for the same data samples of wild-type EGFP-FMBP-1 measured in PSG cells. Each of the 28 samples could be fit well with this fitting model. However, one sample (the 29th measurement sample in Table S1 and S2) could not be fit well, resulting in very few component ratios of the 3rd component, which is impossible to consider real value. Thus, we excluded the one datum from the calculation of average diffusion parameters as shown in the table above.
